# Casposons – silent heroes of the CRISPR-Cas systems evolutionary history

**DOI:** 10.17179/excli2022-5581

**Published:** 2023-01-05

**Authors:** Paulina Smaruj, Marek Kieliszek

**Affiliations:** 1 Department of Quantitative and Computational Biology, University of Southern California, Los Angeles, CA 90089, United States of America; 2 College of Inter-Faculty Individual Studies in Mathematics and Natural Sciences, University of Warsaw, 02-097 Warsaw, Poland; 3 Department of Food Biotechnology and Microbiology, Institute of Food Sciences, Warsaw University of Life Sciences-SGGW, Nowoursynowska 159 C, 02-776 Warsaw, Poland

**Keywords:** Casposons, Cas1, mobile genetics elements, CRISPR-Cas

## Abstract

Many archaeal and bacterial organisms possess an adaptive immunity system known as CRISPR-Cas. Its role is to recognize and degrade foreign DNA showing high similarity to repeats within the CRISPR array. In recent years computational techniques have been used to identify *cas1* genes that are not associated with CRISPR systems, named *cas1-solo*. Often, *cas1-solo* genes are present in a conserved neighborhood of PolB-like polymerase genes, which is a characteristic feature of self-synthesizing, eukaryotic transposons of the Polinton class. Nearly all *cas1*-*polB* genomic islands are flanked by terminal inverted repeats and direct repeats which correspond to target site duplications. Considering the patchy taxonomic distribution of the identified islands in archaeal and bacterial genomes, they were characterized as a new superfamily of mobile genetic elements and called casposons. Here, we review recent experiments on casposons' mobility and discuss their discovery, classification, and evolutionary relationship with the CRISPR-Cas systems.

## Mobile Genetic Elements Integrated into Genome

Mobile genetic elements (MGEs) are DNA fragments encoding proteins that determine their own mobility. The transfer of a given element can take place both between genomes (intercellular mobility) and within the genome of one individual (intracellular mobility). The first - referred to as horizontal gene transfer (HGT) - takes three main forms: conjugation, natural transformation, and transduction (Frost et al., 2005[15]). In turn, multiple specialized types of recombination, usually specifically used by particular MGE families, contribute to intracellular mobility. Mobile genetic elements combine the described transfer strategies, aiming at maximum propagation in the genomes of as many hosts as possible. For example, Integrating Conjugating Elements (ICEs) encode phage-like integrases and other proteins necessary for excision and conjugation (Johnson and Grossman, 2015[19]), while transposons have the ability to "jump" between replicons, so recombination within a plasmid or prophage allows them to invade new hosts *via* HGT (Frost et al., 2005[15]).

The genomes of most bacteria, archaea and eukaryotes contain a variety of integrated mobile genetic elements. Proviruses, ICEs, transposons, integrating plasmids, introns constantly rearrange the chromosomes of organisms within each of the domains of life. It is estimated that half of human genes are derived from transposable elements (TEs) (Lander et al., 2001[33]). Mobile genetic elements are less common among prokaryotes due to the severe pressure that limits their number. Nevertheless, the prophages themselves may constitute up to 20 % of the genome of certain species of bacteria (Casjens, 2003[10]).

## MGE - Good or Bad News?

The presence of mobile genetic elements has two main consequences at the molecular level. First, mobile genetic elements may carry accessory gene cargos that confer a beneficial phenotype to their hosts (Frost et al., 2005[15]). In the population of bacteria exposed to antimicrobial agents, obtaining, transferring, and spreading antibiotic resistance genes is a mean consequence of genetic transposition (Alekshun and Levy, 2007[1]). Molecular analyses suggest that the multidrug resistant *Staphylococcus aureus *arose through the mobilization of resistance genes that were previously present in the bacterial global gene pool (Ramsay et al., 2016[52]). The pressure of globally used antibiotics has led to the proliferation of mobile genetic elements carrying an additional charge in the form of resistance genes (Partridge et al., 2018[49]).

Another example of MGE-mediated phenotypes is virulence (Frost et al., 2005[15]). Profages are one of the main sources of genetic diversity between strains of many pathogens, including *Escherichia coli *(Ohnishi et al., 2001[47])*, Staphylococcus aureus* (Rahimi et al., 2012[51])*, Streptococcus pyogenes* (Banks et al., 2002[4])*, Salmonella enterica* (Cooke et al., 2008[11]). Phages may encode strong extracellular toxins, effector proteins, adhesins, and a range of enzymes such as superoxide dismutase, staphylokinase, phospholipase, DNase (Brüssow et al., 2004[7]).

However, there is some risk involved in the process of acquiring mobile genetic elements. Insertion close to or within the primary metabolism gene may lead to significant changes in its activity: from complete transcription suppression to disturbance of alternative splicing and incomplete protein activity. Moreover, the transposable elements can carry silencers, isolators and other *cis *regulatory elements that modify the expression of genes several thousand base pairs away, and the recognition sites for proteins interacting with DNA. The second consequence is the possibility of recombination between two different *loci*. The effects could be more or less dramatic: from small-scale inversions to rearrangements of entire chromosomes, including deletions, duplications, and translocations (Hua-Van et al., 2011[18]).

## Diversity of Mobile Genetic Elements

In the early 1950s, the existence of the first mobile genetic elements - transposable elements (TEs), was reported to the scientific community. The first MGEs were called pictorially "jumping genes*"* (McClintock, 1950[42]). They have been divided into two classes due to the transposition mechanism (Finnegan, 1989[13]). Class I contains elements transposing by reverse transcription (retrotransposons) with RNA as an intermediate, class II - direct from DNA to DNA. TEs belonging to the second group are often referred to as DNA transposons (Finnegan, 1989[13]).

Retrotransposons are not the only mobile genetic elements that use an RNA intermediate to propagate themselves. Introns within groups I and II constitute another example. These MGE families are able to self-splice due to the autocatalytic properties of ribonucleic acids (Finnegan 1989[13]). Group I introns have been located in the genomes of algae, fungi, lichens, lower eukaryotes, and also among representatives of prokaryotes (Raghavan and Minnick 2009[50]). Group II introns, on the other hand, have been identified in the genes encoding proteins, tRNA, and rRNA in the genomes of numerous bacteria. Among eukaryotes*, *their distribution is limited to mitochondria, and chloroplasts (Marcia et al., 2013[40]). It is widely recognized that group II introns are not present in the nuclear genes, leading to interesting implications in an evolutionary context. The fact that group II introns are present only in mitochondrial, chloroplast and bacterial genomes is considered a strong argument supporting the theory of endosymbiosis (Koonin, 2006[26]). It is hypothesized that the ancestor of eukaryotic cell surrounded its genetic material with a membranous structure to counteract the invasion of its endosymbionts' group II introns (Martin and Koonin, 2006[41]).

A non-trivial role in the evolution of bacterial genomes was played by the mild bacteriophages whose genetic material can integrate into the host's genome (Klimenko et al., 2016[25]). The lysogenic conversion, i.e., changing the phenotype of a harmless strain into a virulent, is one of the possible effects of phage integration. Shiga toxin in *Escherichia coli *O157: H7 (Ohnishi et al., 2001[47]) and cholera toxin in *Vibrio cholerae* (Waldor and Mekalanos, 1996[59]) are examples of virulence factors encoded within prophages. A dozen or even a several dozen prophages can be integrated into a single bacterial genome and share a high degree of sequence homology what enables large-scale recombination and genome rearrangements (Fortier and Sekulovic, 2013[14]). Prophages have a significant impact not only on bacterial virulence and antibiotic resistance, but also on the biofilm formation by, among others, *Streptococcus pneumoniae *(Carrolo et al., 2010[8]).

The reader should bear in mind that mobile genetic elements have a synergistic impact on bacterial genomes. Widespread horizontal gene transfer, Integrating Conjugation Elements, phage-like plasmids, and phages with high similarity to sequenced plasmids blur the boundaries of bacterial cell walls, enabling rapid and effective invasion of prokaryotic genomes. Due to a great number and enormous diversity of the identified mobile genetic elements, we still cannot be sure what part of the MGE iceberg is visible to us.

## Polintons

Polintons (or *Mavericks*) are a group of eukaryotic transposons discovered and characterized with bioinformatics tools. The name "polintons" comes from DNA polymerase and integrase - enzymes encoded by all the elements belonging to this group (Kapitonov and Jurka 2005[21]). The size of the polintons ranges from 15-20 kb and they are flanked by inverted terminal repeat sequences (TIRs). Apart from two universal genes for this group: DNA polymerase from the family B (*polB*) and an integrase resembling those encoded by retroviruses (*int*), most polintons encode ATPases and proteases highly similar to the adenoviral ones (Kapitonov and Jurka, 2005[21]). 

What is particularly interesting, genes coding for capsid proteins as well as enzymes required for their production and cleavage were identified within the polintons (Krupovic et al., 2014[29]). Following this discovery, the hypothesis of the polintons' "dual life" as both transposons and viral particles gained popularity and led to the renaming of these transposable elements to polioviruses (Krupovic et al., 2014[29]). In 2015 metagenomic analyses resulted in the identification of viruses whose genetic architecture significantly resembles the typical for polintons' genes pattern. For this reason, they have been abbreviated as PLV (Polinton-like viruses) (Yutin et al., 2015[64]).

## How to Protect Yourself against the Invasion of Mobile Genetic Elements?

Faced with the continual invasion of mobile genetic elements, prokaryotes have evolved many mechanisms that defend the integrity of their genomes. Acquired immunity, until recently considered a typical animal trait, is common in bacteria and archaea as well. It consists of two key components: (i) CRISPR (*clustered regularly interspaced short palindromic repeats*) - grouped regularly interrupted short palindromic repeats, and (ii) associated proteins (*CRISPR-associated proteins*) - abbreviated as Cas (Sorek et al., 2013[55]). The defence function of CRISPR-Cas systems is the specific degradation of foreign nucleic acid. Specificity is provided by unique spacer fragments (located at CRISPR *loci*) homologous to the DNA of viruses and plasmids (Westra et al., 2012[61]).

CRISPR-Cas systems can be divided into 3 stages:

1. Adaptation - insertion of foreign DNA fragments as spacers in the CRISPR *locus *(Westra et al., 2012[61])*. *The reaction is carried out by a complex of two conserved proteins - Cas1 and Cas2 (Sorek et al., 2013[55]).
2. Expression - the CRISPR *locus *is transcribed into long precursor RNA transcript (pre-CRISPR RNA) which is further processed by specific endonucleases into mature, short cr-RNAs (Sorek et al., 2013[55]).
3. Interference - degradation of foreign nucleic acid (complementary to crRNA) by the endonucleic machinery of Cas proteins (Westra et al., 2012[61])*.*

## Cas1-solo

*Cas* genes not embedded within the canonical CRISPR *loci *were reported for the first time by Makarova et al. (2013[38]). Cas1-solo, as their products came to be termed, form two separate clades of a phylogenetic tree based on the sequence similarity to Cas1. The first clade has been identified in the archaeal order *Methanomicrobiales*, and there are no indications suggesting horizontal gene transfer of those genes (Makarova et al., 2013[38]). In turn, the distribution of Cas1-solo from the second clade is patchy: they have been identified in the class *Methanomicrobia*, several representatives of Thaumarchaeota, and in the euryarcheon *Aciduliprofundum boonei*. It is noticeable that the second group of the *Cas* genes unrelated to the CRISPR *loci * occur in a conserved neighborhood of the family B DNA polymerase (*polB*), which is encoded by polintons, HNH nuclease, and proteins containing the HTH domain (*helix-turn-helix*) (Makarova et al., 2013[38]).

One year later, Krupovic et al. (2014[31]) posed a daring hypothesis suggesting that the CRISPR systems-associated *cas1* genes originated from the recently reported *cas1*-solo genes (Krupovic et al., 2014[31]). The multiple sequence alignment of Cas1 protein sequences showed that none of the first clade members possess all the conserved amino acids necessary for the full enzymatic activity of the Cas1 endonuclease. In contrast, within the second Cas1-solo group, the conservation of all four catalytic residues has been revealed (E141, H208, D218 and D221 in *Escherichia coli* Cas1) (Babu et al., 2011[3]; Krupovic et al., 2014[31]). Consequently, the second clade of the stand-alone *cas1* became the main object of a research focus.

Since the second group of the* cas1*-solo genes occurs in a conserved neighborhood of the family B DNA polymerase (*polB*) (Makarova et al., 2013[38]), other prokaryotic genomes were searched for close co-occurrence of those genes. As a result, 19 genomic islands (from ~ 8 kb to ~ 20 kb long) were identified within genomes of, among others, *Nitrosomonas*, *Streptomyces*, and *Henriciella* (Krupovic et al., 2014[29]).

## How Casposons were Discovered?

Another Krupovic hypothesis (Krupovic et al., 2014[31]) assumed that the islands containing *cas1* and *polB* are indeed integrated mobile genetic elements analogous to the eukaryotic, self-synthetizing polintons. Since a characteristic feature of the DNA transposons is the presence of the TIR sequences (Terminal Inverted Repeats, inverted repeated sequences recognized by transposase) and TSDs (Target Site Duplications) (Jurka et al., 2007[20]), analogous indications of recent mobility have been searched around the stand-alone *cas1* genes. Both TIRs and shorter sequences corresponding to TSDs were identified in the vicinity of almost all *cas1*-*polB* islands (Krupovic et al., 2014[31]).

Intriguingly, no conserved transposase- or recombinase-coding genes have been found within the *cas1-polB* islands. The only enzyme encoded in all the elements that is capable of the DNA fragments integration is the Cas1 endonuclease. Krupovic et al. described the *cas1-polB* islands as a new group of self-synthetizing prokaryotic transposons and called them casposons (Krupovic et al., 2014[31]).

## Mobility of Casposons

Transposons are usually present in more than one copy in a single genome. The exception that proves the rule is the Tn7 family. The formation of tandemly arranged Tn7 transposons islands is a result of multiple integrations into one target site (Parks and Peters, 2009[48]). 

Most of the casposons identified by Krupovic et al. (2014[31]) are present in one copy per genome. However, in the *Methanolobus psychrophilus* R15, two adjacent casposons have been identified. In this specific case, coexistance of mobile genetic elements in close vicinity can be explained by rearrangements within the chromosome and is not synonymous with two integration events into one target site. Furthermore, in the *Methanococcoides burtonii* DSM 6242 three closely related casposons (MetBur-C1, -C2 and -C3) have been found, although the latter is probably inactive due to amber mutations in two genes (Krupovic et al., 2014[31]).

The potential mobility of casposons was deduced from the patchy character of *cas1-polB* islands distribution in prokaryotic genomes. Nevertheless, a comparative genomics analysis of *Methanosarcina mazei* strains showed indications of their recent activity. It constitutes strong evidence that at least some of the casposons are transposable (Krupovic et al., 2014[31]).

## Classification of Casposons

Considering phylogenetic analyses, comparative genomics, and distribution among prokaryotes, casposons were divided into four families (Krupovic et al., 2016[32], 2017[30]). There are significant differences among the set of genes identified within the *cas1-polB *islands, and the genetic organization can vary greatly between families (Krupovic et al., 2014[31], 2016[32]). Interestingly, many casposon-encoded nucleases and helicases are commonly found in the CRISPR-Cas systems, including homologs of the Cas4 nuclease (Krupovic et al., 2014[31]). Most of the additional proteins of prokaryotic adaptive systems belong to the Cas4 family (Koonin and Krupovic, 2014[27]). It has been shown that the Cas4 nucleases play a crucial role in the process of PAM (Protospacer Adjacent Motif) recognition and functional spacers integration during the CRISPR adaptation phase (Kieper et al., 2018[23]; Shiimori et al., 2018[53]).

Casposons classified into the first family occur in members of the *Nitrosopumilus *genus, phylum Thaumarchaeota (Krupovic et al., 2014[31]). Compared to the others, the family 1 casposons are characterized by a compact genetic structure. A set of commonly found genes is limited to *cas1*, *polB*, and three genes encoding products of unknown function (Krupovic et al., 2014[31]). Moreover, the family B polymerase is not closely related to the polymerases found in the other three families. Recent evidence suggests that at some point of evolution *polB* genes were exchanged between casposons and the archaeal viruses His1 and His2 (*Fuselloviridae* and *Pleolipoviridae*) (Makarova et al., 2014[36]).

Casposons classified into the family 2 have been found in the euryarcheal genomes, especially methanogens. An example is the *Methanomassiliicoccus luminyensis* B10, archaea associated with the human intestinal microbiota (Dridi et al., 2012[12]). A characteristic feature of all family 2 members is a C-terminal fusion of the casposase with the protein containing the HTH domain, which has not been found in other casposons. Interestingly, the fusion of a highly similar HTH domain with the family B polymerase has also been identified in the family 2 casposons. Despite significant differences in length (from ~ 6 kb to ~ 16 kb) and in genetic architecture, a set of proteins is universal for this family: Cas1, PolB, endonuclease HNH, and two different proteins with the HTH domain (helix-turn-helix) (Krupovic et al., 2014[31]).

Casposons classified into the third family were found in the genomes of multiple bacteria species, e.g., non-cultured thermophilic *Candidatus "Acetothermum autotrophicum"* (Krupovic et al., 2014[31]). Compared to other families, the genetic architecture is relatively diverse and heterogeneous. Many additional genes and unusual fusions have been identified, like genes encoding HNH and Cas4-like endonucleases, methyltransferases, or Cas1 fused with zinc-binding and helix-turn-helix domain (Krupovic et al., 2014[29]). Considering the phylogenetic analysis of Cas1, the family 3 casposons constitute a separate clade from families 1, 2, and 4 (Krupovic et al., 2014[31], 2017[30]), while on the phylogenetic tree of the family B polymerases, the third family forms a branch departing from casposons of family 2. It has been therefore suggested that casposons appeared as a new group of mobile genetic elements in the archeal genomes and then invaded bacteria *via* HGT (Krupovic et al., 2014[31]). 

The fourth family of casposons was proposed in 2016 when sequences of 62 *Methanosarcina mazei* strains' genomes became available (Krupovic et al., 2016[32]). Interestingly, three different integration sites have been identified within the *M. mazei* genome. Some strains contain multiple casposons creating an array of tandemly integrated elements suggesting that the same target site was used twice by the Cas1 endonuclease. The upper-mentioned facts provide strong evidence that the casposition occurred relatively recently in the evolution of the *M. mazei* species (Krupovic et al., 2016[32]). Again, considerable attention should be received by the fact that some *M. mazei *strains possess both the family 4 and family 2 casposons (Krupovic et al., 2017[30]).

## Enzymatic Characterization of the Stand-alone Casposase

The CRISPR-Cas system's adaptation stage is a complex, a multistep process. The key role in the spacer acquisition is played by a protein complex Cas1-Cas2 (Nuñez et al., 2015[46]). The Cas2 dimer joins two Cas1 dimers to form a heterohexamer (Nuñez et al., 2014[45]). Both proteins are universally conserved across bacterial and archaeal CRISPR-Cas systems (Amitai and Sorek, 2016[2]). As may be expected, mutants encoding a catalytically defective Cas1 protein are unable to introduce new spacer fragments into the CRISPR matrix (Nuñez et al., 2014[45]). Despite the endonuclease activity of Cas2 (Nam et al., 2012[43]), its catalytic activity is not necessary for the integration of new sequences into the CRISPR locus* in vivo *(Nuñez et al., 2014[45]). It suggests that the role of Cas2 in the Cas1-Cas2 complex is reduced to the formation of a structural scaffold (Krupovic et al., 2017[30]).

Casposons do not contain conserved genes coding transposases, nor recombinases - determinants of other MGE families' mobility. Consequently, the Cas1 protein is most likely responsible for casposons' transposition due to its endonucleolytic activity necessary to integrate and excise DNA fragments from the genome (Krupovic et al., 2014[31]). So far, no casposon encoding Cas2 has been reported (Krupovic et al., 2017[30]).

Casposase target site preference has already been suggested by Krupovic et al., (2014[31]). Out of 19 *cas1-polB* islands identified by them: three are located at the aEF-2 translation elongation factor gene's 3' end, while five euryarchaeal casposons were found within the 3' region of the tRNA genes (Krupovic et al., 2014[31]). Some of the casposase and the CRISPR-Cas1 biochemical features are compared in Table 1(Tab. 1) (References in Table 1: Béguin et al., 2016[5]; Hickman and Dyda, 2015[16]; Kim et al., 2013[24]; Krupovic et al., 2014[31], 2017[30]; Nuñez et al., 2014[45], 2015[46]; Silas et al., 2016[54]; Wiedenheft et al., 2009[62]).

First studies of the endonuclease properties of the stand-alone Cas1 focused on the casposase purified from *Aciduliprofundum boonei* (Hickman and Dyda, 2015[16]). Research has consistently shown that both short oligonucleotides and 2.8 kb-long DNA fragments could be integrated by casposase if they are flanked with terminal inverted repeats (TIRs). The integration results in the generation of 15-bp-long target site duplications (TSDs). The first experiments suggested also that Cas1 has no sequence specificity, and the integration site is random (Hickman and Dyda, 2015[16]). However, this surprising result can be explained by the lack of reconstructed integration target site in a casposase's substrate. 

While examining the same enzyme, Béguin et al. (2016[5]) came to the opposite conclusions. In the presence of the integration target site, the Cas1 casposase of *A. boonei* shows a high preference. An artificial casposon carrying a kanamycin resistance gene and flanked with TIR sequences was integrated (often in tandem) into the reconstructed target site at the 3′ end of the tRNA-Pro gene generating short duplications at the ends (TSDs). 

In 2019, the DNA motif recognized by the *A. boonei *casposases has been reported (Béguin et al., 2019[6]). The critical residues for the casposon integration are five terminal nucleotides at the 3' casposon's end. This is also true for the Cas1 nuclease isolated from *Nitrosopumilus koreensis* despite being classified into different family of caposons (Béguin et al., 2019[6]).

Interestingly, sequences recognized by the Cas1 endonuclease during the protospacer and casposon integration share some genetic characteristics (Krupovic et al., 2017[30]). In both cases, the target site consists of two parts: 

(i) a sequence that is duplicated (the repetitive sequence in the CRISPR locus, and the casposons' TSDs) (Krupovic et al., 2017[30]),

(ii) the upstream sequence that determines the integration target site (the leader sequence in the CRISPR locus, and the 18-nucleotide segment encoding the TψC loop in tRNA-Pro in the case of casposase *A. boonei*) (Béguin et al., 2016[5]; Krupovic et al., 2017[30]).

Furthermore, the casposon integration strictly depends on the distal parts of the terminal inverted repeats flanking a mobile element (Hickman and Dyda 2015[16]), while in the *E. coli* CRISPR-Cas system, the protospacer integration specificity is determined by the PAM sequence (Westra et al., 2013[60]).

Due to the nature of the integration target site, the CRISPR locus has a repeating pattern. Spacers are separated by short repeats, which implies the integration site reconstruction after each spacer acquisition event (Amitai and Sorek, 2016[2]). Given the similarities between the sequences recognized by the CRISPR-Cas1 and casposase, one can expect the existence of multiple casposons integrated head-to-tail. Indeed, tandem casposons separated only by TSD sequences have been identified in the genomes of certain archaea (e.g., *Methanosarcina* sp.) (Krupovic et al., 2016[32]).

## Mechanism of Action of the Stand-alone Cas1

The mechanism of the casposase's action has not been in detail explained yet. Nevertheless, based on the similarities between the CRISPR-Cas1 and casposase target sites, the hypothetical model of casposon integration was derived.

Detailed studies of the enzymatic activity were mainly focused on the *A. boonei *Cas1, which recognizes the palindromic repeat forming the TψC loop in tRNA-Pro (Béguin et al., 2016[5]). According to the proposed model, the border of the TSD and the tRNA-Pro gene is attacked by the casposon's 3'-OH end resulting in the half-site intermediate formation. Afterwards, the second nucleophilic substitution occurs at the other TSD's side. It is likely that the distance between casposase dimer active sites determine where the second attack appears. As a result of the casposon integration, single-stranded breaks in DNA are generated. According to the Béguin's model, gaps are filled in creating target site duplications (TSDs), although the molecular mechanisms fixing DNA breaks has not been described so far (Béguin et al., 2016[5]).

Recent experimental data indicate that the casposase prefers single-stranded DNA as a substrate (Hickman et al., 2020[17]). This fact, together with the conserved presence of the DNA polymerase gene in all identified casposons, suggest that the replication of a single-stranded DNA play a key role in the casposons mobility. It is very likely that the Béguin's model (Béguin et al., 2016[5]) will be revised in the future.

## Casposase Structure

In 2020 the detailed characterization of the *M. mazei* casposase's biochemical properties was published together with the structure of the *M. mazei* casposase in complex with branched DNA (Hickman et al., 2020[17]). The solved complex represents the effect of a single-stranded casposon insertion into a specific sequence located at the 3' end of the tRNA-Leu gene. Surprisingly, when bounded to its target site, the Cas1 endonuclease from *M. mazei* forms a homotetramer (Hickman et al., 2020[17]). The active complex can integrate not only casposons with single-stranded 3'-ends, but also ssDNA and ssRNA substrates. The results indicate that the integration occurs precisely into the specific target site. Moreover, the presence of a specific sequence motif located upstream of the potential integration site is required by the *M. mazei *Cas1 casposase, similarly as the insertion of spacer sequences into the CRISPR locus relies on the presence of a specific leader sequence (Hickman et al., 2020[17]).

Based on the gathered data, the authors proposed a scenario for the Cas protein complexes evolution (Hickman et al., 2020[17]). According to the model, the Cas1 protein lost the ability to interact with DNA in the tetrameric form, when the binding between Cas1 and Cas2 components became preferable than the Cas1-Cas1 interaction. It has been suggested that the Cas2 dimer present in the CRISPR-Cas system-associated heterohexamers functions as a structural bridge between two separately inactive Cas1 dimers, that orients them towards each other and provides an additional DNA binding surface. The sampling of more specific crRNAs produced on the template of longer spacers might have functioned as an evolutionary driving force behind such a fundamental change in the complex architecture (Hickman et al., 2020[17]).

## Regulation of the Casposons Mobility

Little is known about the molecular mechanisms regulating the casposition. Interestingly, a link between stress conditions and the upregulation of the *cas1*-solo gene transcription in the* M. mazei *Gö1 strain has been recently reported (Ulbricht et al., 2020[58]). 

A few years ago, the relationship between the CRISPR-Cas system induction and high-salt condition was observed (Nickel et al., 2013[44]). The recent identification of the MM_0565 protein from the *M. mazei* Gö1 strain shed a new light on this mysterious association (Ulbricht et al., 2020[58]). Transcriptomic analyses indicate that the MM_0565 overproduction results in the upregulation of four casposase genes within the CRISPR-Cas I-B operon (*cas8b*, *cas7*, *cas5*, and *cas3*). At the same time, transcription of other casposase genes (*cas6b*, *cas1*, *cas2*, *cas4*) whose products play a role at the adaptation stage and in the crRNA maturation are not significantly affected (Ulbricht et al., 2020[58]). Surprisingly, the mRNA level of a stand-alone *cas1* gene, that was previously characterized as a part of a *M. mazei* caposon, is enhanced by 36-fold when the MM_0565 protein is overproduced. Considering the stress-induced mobility of transposable elements (Casacuberta and González, 2013[9]) and the high stability of MM_0565 at high salinity *in vivo*, the authors suggested that the MM_0565 protein may be involved in the regulation of casposon mobility under osmotic stress (Ulbricht et al., 2020[58]).

## The Role of Casposons in the Evolution of CRISPR-Cas Systems

The hypothesis of the emergence of the CRISPR-Cas systems in the archaea domain was presented even before the identification of casposons (Makarova et al., 2011[35]). Later, a key role of casposons in the evolution of the prokaryotic adaptive systems was suggested multiple times (Krupovic et al., 2014[31], 2016[32], 2017[30]; Béguin et al., 2016[5]).

However, it should be noted that back in 2014, both considering casposons a new MGE group and ancestors of the CRISPR-Cas systems were attractive, albeit daring, hypotheses. The article by Krupovic et al. (2014[31]) aroused keen interest in the scientific community, even though the discovery of casposons was based only on *in-silico* results. The formulated hypothesis linking the origin of the CRISPR-Cas systems with the identified *cas1-polB* islands pushed the research on casposons towards the experimental stage.

Undoubtedly, the Krupovic's hypothesis might have been met with skepticism. Experimental studies concern a very narrow pool of Cas1 proteins - biochemical data are available only on the casposases from *A. boonei*, *M. mazei*, and *N. koreensis*. Based on the properties of the recombination reaction carried out by the *A. boonei* Cas1 enzyme, a model of casposon integration was proposed, in which the key role is played by the palindromic repeat encoding the TψC loop in tRNA-Pro molecule. However, there are casposons whose conserved integration site is not localized in the tRNA genes neighborhood. 

Doubts may also be raised by the bold conclusions drawn from the Cas1 phylogenetic analysis that is not used in the classification of CRISPR-Cas systems. The genetic architecture of the prokaryotic adaptive systems is highly complex, and individual modules show a certain evolutionary independence (Koonin and Makarova, 2019[28]). Even if we assume that the Cas1 protein phylogenesis reflects the relationship between casposon families and the CRISPR-Cas systems, the position of the root in the Cas1 tree is undefined. One may say that placing the evolution of Cas1 endonucleases into the dimension of time is unjustified.

On the other hand, the CRISPR-Cas is not the first defense system that evolutionary origin is linked to mobile genetic elements. The V(D)J recombination, that plays a crucial role in the specificity of the vertebral adaptive immune system, creates an enormous variability of antibodies, T- and B-lymphocytes receptors (Tonegawa, 1983[57]). The reaction is catalyzed by the V(D)J recombinase whose main subunits are proteins RAG1 and RAG2 (Swanson, 2004[56]). Recent evidence suggests that both the RAG1 and RAG2 evolved from a single transposase encoded by the *Transib* superfamily transposon (Kapitonov and Koonin 2015[22]), although alternative evolutionary scenario has also been proposed (Yakovenko et al., 2021[63]).

It is true that the origin of the adaptive module of CRISPR-Cas systems from casposons is indicated primarily by the unrooted phylogenetic tree based on the sequence similarity of the Cas1 endonucleases. Nevertheless, the tree branches into two main clades: casposons and Cas1 proteins associated with the CRISPR-Cas systems (Krupovic et al., 2014[29]). Despite the unknown location of the root, such a branching indicates the role of casposons in the evolution of the prokaryotic adaptive system (Koonin and Makarova, 2019[28]).

According to the generally accepted evolutionary scenario, the adaptive module of CRISPR-Cas systems arose from a casposon which acquired other genes in subsequent stages of evolution (Makarova et al., 2014[36]). None of the casposons identified so far encode the Cas2 protein - a key subunit of the adaptive module complex, although other components characteristic to the CRISPR-Cas systems, including the Cas4 nuclease, have been identified within casposons (Krupovic et al., 2014[29]). It is very likely that the first CRISPR-Cas system appeared in the archaeal ancestor as a result of the casposon insertion adjacent to the primary effector module (cascade operon). Later, the loss of one of the TIR sequences resulted in casposon immoblization. Other accessory genes, including *polB*, were lost afterwards (Koonin and Krupovic, 2014[27]).

Initially, it was assumed that the CRISPR-array repeats evolved from inverted repeated sequences (TIRs) flanking casposons (Koonin and Krupovic, 2014[27]). This hypothesis was based on some similarities:

(i) there are casposons with palindromic TIR sequences (Krupovic et al., 2014[29]), 

(ii) some of them share some sequence similarity, and

(iii) the postulated binding preference of the Cas1 protein (Koonin and Krupovic, 2014[27]).

Although the length of inverted repeat sequences (TIRs) can vary greatly (25-602 bp), the most common is ~50 bp (Krupovic et al., 2014[29]) which correlates with the size of the CRISPR repeats (20-50 bp) (Sorek et al., 2013[55]).

Further biochemical and structural studies of both the CRISPR-Cas and casposase systems led to the rejection of the above hypothesis. It was proposed instead that after casposon immobilization, the target site evolved into the repeat sequence of the future CRISPR system (Krupovic et al., 2017[30]). Recent data also suggest the direct evolution of the leader sequence from the casposase recognition site. The next key step in the evolution of the prokaryotic adaptive system was the Cas2 recruitment (Krupovic et al., 2017[30]), followed by the transition from homotetrameric casposase complex to the Cas1-Cas2 heterohexamer (Hickman et al., 2020[17]). Due the homology between Cas2 and some proteins encoded by the toxin-antitoxin systems, it is hypothesized that such a TA module might have been carried by the ancestral casposon (Krupovic et al., 2017[30]). Alternatively, *cas2* gene was acquainted from an independent toxin-antitoxin system (Koonin and Makarova, 2019[28]).

## Conclusion

The variety of mobile genetic elements is astonishing and far from being fully understood. The development of bioinformatics data analysis tools in the last two decades has created opportunity not only to faster and comprehensive research on previously known MGE families, but also to identify a completely new one. We believe that the upcoming years of MGE research will lead to discovery of new casposons, very likely classified to the entirely new casposons families. As just in 2020, a surprisingly diverse group of CRISPR-Cas systems and Cas1 homologues encoded by archaea classified into the Asgard supertype were identified (Makarova et al., 2020[39]). In the same year, an evolutionary classification CRISPR-Cas systems and casposase genes was updated with a numerous new CRISPR-Cas variants and* cas *homologs (Makarova et al., 2020[37]). 

Despite the doubts that might arise regarding both the discovery and further casposons analysis, this superfamily of MGE should not be ignored. Researchers published strong evidence of their recent transposable activity, and the caposase site-specific recombinant activity has been experimentally confirmed. Furthermore, the structure of the *M. mazei* casposase in complex with branched DNA provide additional support for the suggested evolutionary hypotheses.

Casposases constitute an important research object not only because they gave rise to the CRISPR-Cas machinery but also due to their potential application as molecular biology tools. It has been shown that the casposase of *A. boonei* can insert any DNA fragment, including those that are not flanked by TIR sequences. In addition, fusion of the *A. boonei* Cas1 endonuclease with the bacterial Cas9 enables researchers to control the integration site *in vitro*. The results of the first Cas1-Cas9 applications are highly encouraging. Therefore, it cannot be ruled out that the Cas1-Cas9 fusion protein will soon become an RNA-directed genome editing tool (Lau and Bolt, 2021[34]).

## Notes

Paulina Smaruj and Marek Kieliszek (Department of Food Biotechnology and Microbiology, Institute of Food Sciences, Warsaw University of Life Sciences—SGGW, Nowoursynowska 159 C, 02-776 Warsaw, Poland; E-mail marek_kieliszek@sggw.edu.pl) contributed equally as corresponding author.

## Conflict of interest

The authors declare that there is no conflict of interest to disclose, either financial or non-financial. 

## Figures and Tables

**Table 1:**
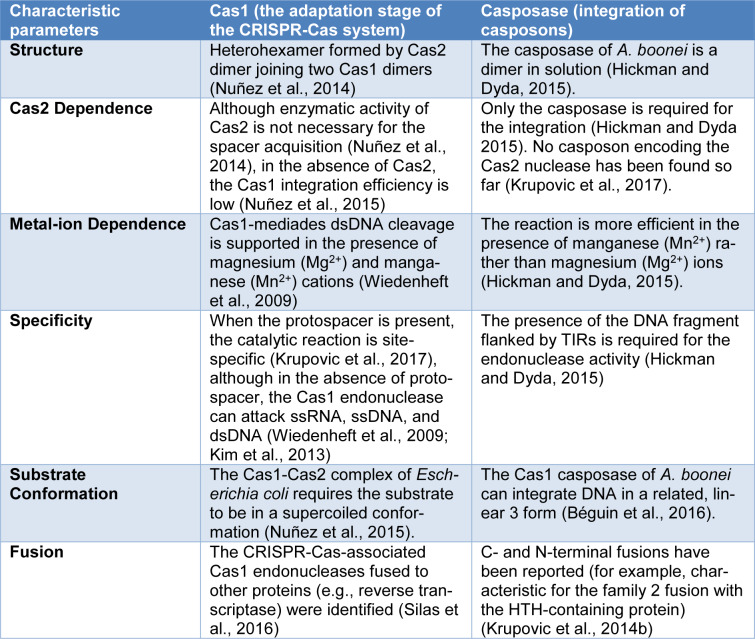
The comparative analysis of the casposases and the CRISPR-Cas1 nucleases
